# Proceedings of the Rochester Hahnemannian Society

**Published:** 1888-10

**Authors:** W. H. Baker

**Affiliations:** Secretary


### PROCEEDINGS OF THE ROCHESTER HAHNEMANNIAN SOCIETY.

The regular monthly meeting of the Rochester Hahnemannian Society was held at the office of Julius Schmitt, M. D., July 17th, Dr. Grant in the chair.

Members present: Drs. Grant, Schmitt, Carr, A. C. Hermance, Baker; Dr. Brownell, of this city, was present as a visitor.

Minutes of the last meeting were read and approved. Sections 156 to 166 were read from the *Organon.*

Dr. Baker—Hahnemann says that in acute diseases an aggravation during the first few hours is generally an excellent indication that the disease will yield to the first dose; this is a sure guide for us not to repeat, but wait for the one dose to act.

Dr. Brownell—Do we not all get cases where there is no aggravation?

Dr Carr—I saw a case last week, a man who had worked all day, had severe headache, dry heat, restlessness, pulse 104, temperature 103°. Prescribed Aconite. On the following day, headache the same, and worse from motion; restlessness gone; disposition to lie still; tongue white; soreness of the diaphragm; worse sitting up with nausea. Gave Bryonia. Twenty-four hours afterward, pulse was 88, temperature 99°; headache continued with many of the same symptoms of the day before; gave Sac. lac., and in twenty-four hours patient was cured. I believe the Bryonia aggravated the case, but as the pulse and temperature were better, I continued Sac. lac., and the case was cured in twenty-four hours.

Dr. Schmitt—I think Aconite was indicated at first; symptoms will change in twelve hours, calling for a new remedy. Bryonia was not indicated at the start. Hahnemann does not say aggravations must come; a case can be cured without any aggravation.

Dr. Carr—I place more dependence upon the aggravation the patients observe.

Dr. Schmitt—Hahnemann says in a medicinal aggravation, some symptoms will be better, so we may know it is an aggravation from the remedy and not the disease that is worse.

Dr. Brownell—When you get a seeming improvement, yet the pulse and temperature are worse, I look upon it as a bad sign.

Dr. Grant—I will give a case where the remedy produced other drug symptoms. A married lady consulted me for a cough that returned every February and lasted until summer or warm weather, which would always relieve, and she was free from the cough until the following February. She had had measles three years before, and had taken cold during that illness. Her symptoms were: *severe racking cough; inclination to vomit during cough; aggravation in the morning; expectoration of yellowish muçus of a sweetish taste.* I gave Stannum$^{200}$, one dose, and told her to come again in one week; the cough was cured, and there has been no return for the past two winters. I asked her when first she began to improve; she said the following morning, after she was in my office, she had the worst coughing spell she ever had, which was followed by profuse expectoration of saltish mucus. It was sweet before, now saltish; after this she improved rapidly.

Dr. A. C. Hermance—Sweetish mucus is characteristic of Stannum.

Dr. Carr—It was fortunate you told her not to come back for one week, for if you had seen her the next morning, you might have been tempted to do something.

Dr. Brownell then read an interesting and instructive

